# Mitoprotective Effects of a Synergistic Nutraceutical Combination: Basis for a Prevention Strategy Against Alzheimer’s Disease

**DOI:** 10.3389/fnagi.2021.781468

**Published:** 2022-02-21

**Authors:** Dona P. W. Jayatunga, Eugene Hone, W. M. A. D. Binosha Fernando, Manohar L. Garg, Giuseppe Verdile, Ralph N. Martins

**Affiliations:** ^1^Centre of Excellence for Alzheimer’s Disease Research & Care, School of Medical and Health Sciences, Edith Cowan University, Joondalup, WA, Australia; ^2^Cooperative Research Centre for Mental Health, Carlton, VIC, Australia; ^3^Faculty of Health and Medicine, School of Biomedical Sciences and Pharmacy, University of Newcastle, Callaghan, NSW, Australia; ^4^Riddet Institute, Massey University, Palmerston North, New Zealand; ^5^Faculty of Health Sciences, School of Pharmacy and Biomedical Sciences, Curtin Health Innovation Research Institute, Curtin University, Perth, WA, Australia; ^6^Australian Alzheimer’s Research Foundation, Ralph and Patricia Sarich Neuroscience Research Institute, Nedlands, WA, Australia; ^7^Department of Biomedical Sciences, Macquarie University, Sydney, NSW, Australia

**Keywords:** mitobiogenesis, mitochondrial dysfunction, mitophagy, reactive oxygen species, synergistic nutraceutical combination, Alzheimer’s disease

## Abstract

Evidence to date suggests the consumption of food rich in bioactive compounds, such as polyphenols, flavonoids, omega-3 fatty acids may potentially minimize age-related cognitive decline. For neurodegenerative diseases, such as Alzheimer’s disease (AD), which do not yet have definitive treatments, the focus has shifted toward using alternative approaches, including prevention strategies rather than disease reversal. In this aspect, certain nutraceuticals have become promising compounds due to their neuroprotective properties. Moreover, the multifaceted AD pathophysiology encourages the use of multiple bioactive components that may be synergistic in their protective roles when combined. The objective of the present study was to determine mechanisms of action underlying the inhibition of Aβ_1–42_-induced toxicity by a previously determined, three-compound nutraceutical combination D_5_L_5_U_5_ for AD. *In vitro* experiments were carried out in human neuroblastoma BE(2)-M17 cells for levels of ROS, ATP mitophagy, and mitobiogenesis. The component compounds luteolin (LUT), DHA, and urolithin A (UA) were independently protective of mitochondria; however, the D_5_L_5_U_5_ preceded its single constituents in all assays used. Overall, it indicated that D_5_L_5_U_5_ had potent inhibitory effects against Aβ_1–42_-induced toxicity through protecting mitochondria. These mitoprotective activities included minimizing oxidative stress, increasing ATP and inducing mitophagy and mitobiogenesis. However, this synergistic nutraceutical combination warrants further investigations in other *in vitro* and *in vivo* AD models to confirm its potential to be used as a preventative therapy for AD.

## Introduction

Alzheimer’s disease (AD) is the most common form of dementia that constitutes approximately 60–80% of clinically diagnosed dementia cases, worldwide (Alzheimer’s Association, 2020). The need for an effective treatment for AD is critical, especially considering the rapidly growing number of people living with the disease and the associated socioeconomic impact (Alzheimer’s Association, 2020). However, there is currently limited success in clinical trials, with most ending in failure (Oxford et al., 2020). Most of the criticisms are attributed to the targeting of the clinical phenotype, where there is existing irreversible synaptic and neuronal degeneration and profound inflammatory changes (Huang et al., 2020). Other than the controversies related to very recently approved drug Aducanumab (Selkoe, 2021), the current situation where there is still no AD-targeted-therapy pending approval soon emphasizes the need for identification of AD preventive strategies. This approach targeting prevention is the most appealing, considering the long prodromal phase of AD, that can span more than 10 to 20 years before clinical signs of AD appear (Villemagne et al., 2013). As illustrated by Hodes et al. (2019), there is an ongoing debate over the use of the terms “prevention” and “risk reduction” in AD (Hodes et al., 2019). However, in either case of preventing or delaying the related cognitive decline, AD onset and progression requires a clinical intervention and alternative, preferably non-pharmacological, options have become increasingly attractive.

Lifestyle is thought to be one of the key modifiable risk factors for AD, with diet playing a major role (Hu et al., 2013). Thus, diet is important as a potential target for the development of preventive strategies for AD (Dadhania et al., 2016). The bioactive components of diets, the nutraceuticals (Kalra, 2003), are being investigated for their capabilities in preventing AD onset and progression (Farías et al., 2014). Accumulating evidence suggests that nutraceuticals, such as polyphenols and specific fatty acids found in fruits, vegetables, herbs, and nuts may slow neurodegeneration and improve memory and cognitive function (Joseph et al., 2009; Mecocci et al., 2014; Keservani et al., 2016). Furthermore, nutraceutical combinations can be the most effective, as they may act synergistically against the multiple aspects of AD pathogenesis. In general, the different phytochemicals in a nutraceutical combination can activate different cellular pathways by improving the bioavailability of active compounds. Thus, an advantage of a synergistic nutraceutical combinations is the possibility to reduce doses of individual compounds resulting in toxic effects at minimum (Santana-Gálvez et al., 2019). Therefore, synergistic nutraceutical combinations can be used to formulate novel and effective functional food products with anti-AD properties.

Recently, we identified a three-compound synergistic nutraceutical combination D_5_L_5_U_5_ that exhibited synergistic activity against Aβ_1–42_-induced toxicity in cell culture (Jayatunga et al., 2021). It consisted of three nutraceutical compounds docosahexaenoic acid (DHA), luteolin (LUT), and urolithin A (UA) each at 5 μM concentration. These constituents were considered in deciphering a probable mechanism of action for this nutraceutical combination. Docosahexaenoic acid is reported to modulate mitochondrial biogenesis, which is partially associated with increased mtDNA replication and PGC-1α gene expression in C2C12 muscle cells (Lee et al., 2016) and in C57BL/6J mice (Flachs et al., 2005). There is evidence that DHA promotes apoptosis and autophagy, however, there are no reports to date on its effect on mitophagy, which is the selective autophagy of superfluous mitochondria (Lemasters, 2005; Kubli and Gustafsson, 2012). The protective effects of LUT are associated with up-regulation of autophagy and improvement of mitochondrial biogenesis through Mst1 inhibition (Hu et al., 2016). Furthermore, LUT has exerted neuroprotection by enhancing autophagy and anti-oxidative processes in both *in vivo and in vitro* models of intracerebral hemorrhage (Tan et al., 2020). Luteolin has been recently reported to induce mitochondrial apoptosis in HT29 cells by inhibiting the Nrf2/ARE signaling pathway (Yang et al., 2020). Furthermore, LUT has protected endothelial cells against H_2_O_2_-induced oxidative stress via modulating ROS-mediated P38 MAPK/NF-κB and calcium-evoked mitochondrial apoptotic signaling pathways (Chen et al., 2020). However, evidence is scarce that LUT improves mitochondrial dynamics and mitophagy. It is increasingly identified that UA restores mitochondrial dysfunction in the aging skeletal muscle through up-regulation of genes associated with mitochondrial biogenesis, mitophagy and fatty acid oxidation (Yang, 2019). Recently, it has been proven in C57BL/6 male mice fed on high fat diet that UA resulted in elevated mitochondrial biogenesis in the liver (Toney et al., 2019) along with inducing mitophagy (Ryu et al., 2016). One study reported that mitophagy is not induced by UA in both ischemia/reperfusion−injured neuronal cells and mice (Ahsan et al., 2019). Therefore, further studies are still needed to confirm the mitophagy-inducing potential of UA. Taken collectively, these previous reports on mitoprotective effects of DHA, LUT, and UA provide further motivation to investigate if the beneficial effects still exist when the same are combined in low concentrations.

The present study was undertaken to evaluate the *in vitro* pathways D_5_L_5_U_5_ take to protect against Aβ_1–42_-induced toxicity. For comparison, the components of this combination, DHA, LUT, and UA were also assessed for their ability to protect from Aβ_1–42_-induced toxicity.

## Materials and Methods

### Materials

cis-4,7,10,13,16,19-Docosahexaenoic acid (DHA: D2534), LUT (L9283), UA (SML1791), N-acetyl L-Cysteine (NAC: A9165), Resveratrol (RES), Bafilomycin A (Baf A: B1793), sodium docecyl sulfate (SDS), Triton X-100, and dimethyl sulfoxide (DMSO) were obtained from Sigma Aldrich, United States. BE(2)-M17 cells (ATCC^®^ CRL2267™) were purchased from American Type Cell Culture Collection (ATCC, Manassas, VA, United States). All cell culture reagents including Dulbecco’s Modified Eagle Medium (DMEM), Ham’s F12 medium, Hank’s balanced salt solution (HBSS), fetal calf serum (FCS) and Trypsin-EDTA (0.5%) were purchased from GIBCO by Life Technologies (United States). Human Aβ_1–42_ peptides were synthesized, purified and characterized by high pressure liquid chromatography (HPLC) and mass spectrometry (MS) by The ERI Amyloid Laboratories LLC, United States. Anhydrous DMSO was purchased from Molecular Probes by Life Technologies (United States). CellTiter-Glo luminescent cell viability assays were purchased from Promega, Wisconsin, United States and 2′,7′-dichlorofluorescin diacetate (DCFDA) intracellular ROS assay kits (ab113851) were obtained from Abcam, Cambridge, United Kingdom. Bolt 4–12% Bis-Tris plus gels, gel running MES [2-(N-morpholino) ethanesulfonic acid] buffer, 4X Bolt Lithium dodecyl sulfate (LDS) sample buffer NuPAGE, 10X Bolt sample reducing agents, Novex Sharp pre-stained protein standards were purchased from Life Technologies, United States. Primary antibodies, p62, PINK1, TOMM20, TIMM23, FIS1, and PGC-1α and goat anti-rabbit Alexa Fluor488 secondary antibody were obtained from Abcam, Cambridge, United Kingdom. Primary antibodies, LC3B, MFN2, NDP52, OPTN and OPA1 were obtained from Cell Signaling. For Western blotting, trans blot turbo mini nitrocellulose transfer packs were purchased from Biorad, United States. Secondary antibodies, anti-mouse and anti-rabbit immunoglobulin G (IgG) horse radish peroxidase (HRP) linked whole antibodies and enhanced chemiluminescence (ECL) kits were obtained from GE Healthcare, United Kingdom.

### Cell Culture

Human neuroblastoma BE(2)-M17 cells were maintained in T75 culture flasks containing 15 mL of DMEM/F12 (1:1 ratio) growth media supplemented with 10% (v/v) FCS and placed in a humidified incubator with 5% CO_2_/95% air at 37°C. Upon reaching about 80% confluency, the cells were sub-cultured on to fresh cell culture flasks. For all cell culture experiments, passage number did not exceed 30.

### Aβ_1–42_ Peptide Preparation

Oligomeric Aβ_1–42_ was prepared according to the method of Stine et al. (2011) with some modifications. Briefly, synthetic human Aβ_1–42_ peptide of 4.5 mg was dissolved in 1000 μL HFIP solution and incubated for 30 minutes at room temperature (RT). The Aβ_1–42_ dissolved HFIP solution was aliquoted using positive displacement pipetting, into 10 microcentrifuge tubes each containing 0.45 mg Aβ_1–42_ peptide. The aliquots were then air-dried in a fume cupboard overnight at RT. The tubes containing the peptide films were further dried in a Savant vacuum concentrator (–80 °C). The Aβ_1–42_ films were then stored at –30 °C in a sealed container with desiccant. All Aβ_1–42_ peptide solutions were freshly prepared for each experiment. The 0.45 mg peptide film in the tube was dissolved in 20 μL of anhydrous DMSO (Molecular probes). The solution was briefly vortexed, then sonicated in a water bath for 10 minutes with temperature controlled by adding ice so as not to exceed RT. Then, 980 μL of ice-cold F12 media (without phenol red) was added. The solution was then briefly vortexed and incubated for 24 hours at 4 °C to be used in Aβ_1–42_ toxicity experiments the next day.

### Treatments

In all treatments, the cells were pre-treated with the compound combination D_5_L_5_U_5_ (DHA 5 μM, LUT 5 μM, and UA 5 μM) and the individual compounds, DHA (30 μM), LUT (20 μM), and UA (30 μM) for 24 h. These concentrations of DHA, LUT, and UA were selected based on our previous studies (Jayatunga et al., 2021). Resveratrol (RES) was used as a positive control in all experiments as it is a known agent of inducing mitochondrial dynamics, mitophagy and mitobiogenesis. N-acetyl cysteine (NAC) is an antioxidant that was used as a reference compound for ROS analysis. The concentrations of RES and NAC used in the experiments were based on the available literature (Mitsopoulos and Suntres, 2011; Gueguen et al., 2015).

In all experiments, after pre-treatment with the compounds was followed by 20 μM oligomeric Aβ_1–42_ treatment for 16 h unless otherwise specified. This time period was selected based on our previous findings that mitophagy is diminished after 16 h of 20 μM Aβ_1–42_ treatment in BE(2)-M17 cells (Jayatunga et al., unpublished). All treatments were carried out with appropriate Aβ_1–42–_treated and vehicle-treated controls.

### DCFDA Assay

The BE(2) M17 cells seeded in 96 well plates at a density of 25 × 10^3^ cells/well. After 24 h, the cells were pre-treated with the compounds for 24 h. The cells were then exposed to 20 μM Aβ_1–42_ for 4 h in the incubator at 37°C and intracellular ROS levels were determined using the DCFDA assay. Aβ_1–42_ incubation of 4 h was based on our previous work (Jayatunga et al., unpublished). Briefly, 45 min prior to completion of the treatment, the cells in color-free treatment media (100 μL per well) were overlaid with 100 μL of warm 50 μM DCFDA solution [diluted from the DCFDA stock (20 mM) using 1X wash buffer]. The microplate was incubated for 45 min at 37°C and fluorescence readings (Relative Fluorescence Units: RFU at excitation 485 nm/emission 535 nm) were obtained at excitation 485 and 535 nm using a Perkin Elmer EnSpire multi-mode plate reader.

### CellTiter Glo Assay

The BE(2)-M17 cells were seeded in 96-well plates at a density of 15 × 10^3^ cells/well. After 24 h, the cells were pre-treated with the compounds for 24 h. After exposure of 20 μM Aβ_1–42_ for 4, 8, 16, 24, 48, and 72 h, the CellTiter Glo assay was used to determine relative ATP levels. Briefly, cells were placed at RT for 30 min and then lysed by adding 100 μL of ATP-releasing reagent. The lysates were incubated with the luciferin substrate and luciferase enzyme in the dark for 10 min to stabilize the luminescence signal. The luminescence (RLU) was measured using a Perkin Elmer EnSpire multi-mode plate reader.

### Western Blot Analyses

The BE(2)-M17 cells were seeded in T25 cell culture flasks at a density of 1 × 10^6^ cells per flask and grown for 24 h. Test samples were prepared by compound pre-treatments followed by 20 μM oligomeric Aβ_1–42_ treatments for 16 h. Additionally, for p62, NDP52 and OPTN, Bafilomycin A (Baf A) was added to the cell flasks after 15 h, i.e., 1 h prior to reaction completion. The cells were lysed in precooled lysis buffer (SDS: Triton X-100, 7:3, protease inhibitor, PBS) and protein concentrations were determined using a BCA protein assay kit. Protein samples were separated on 4–12% Bis-Tris gels by polyacrylamide gels electrophoresis (SDS-PAGE) and transferred onto nitrocellulose membranes. Then, membranes were blocked in 5% non-fat milk for 30 min at room temperature and incubated with primary antibodies (rabbit monoclonal MFN2 antibody; 1:1,000, rabbit monoclonal OPA1 antibody; 1:2,000, rabbit monoclonal FIS1 antibody; 1:5,000, mouse monoclonal p62 antibody; 1:2,000, rabbit polyclonal LC3B antibody; 1:1,000, rabbit polyclonal PINK1 antibody; 1:1,000, rabbit monoclonal NDP52 antibody; 1:1,000, rabbit monoclonal OPTN antibody; 1:500, rabbit polyclonal TIMM23 antibody; 1:200, mouse monoclonal PGC-1α antibody; 1:1,000) overnight at 4°C. Membranes were then washed and incubated with HRP-conjugated goat anti-rabbit and HRP-conjugated goat anti-mouse (1:5,000) secondary antibodies for 1 h at room temperature followed by development using ECL detection. The protein bands were normalized against GAPDH, using ImageLab program (Biorad; v. 6.0.1).

### Statistical Analysis

All results were expressed as mean ± standard deviation (SD) from four (*N* = 4) independent experiments. Statistical significance was determined by one-way ANOVA and Tukey’s *post hoc* test in SPSS v25. Significance was defined as *P* < 0.05.

## Results

### D_5_L_5_U_5_, DHA, Luteolin, and Urolithin A Reduce Aβ_1–42_-Induced ROS Levels

**FIGURE 1:**
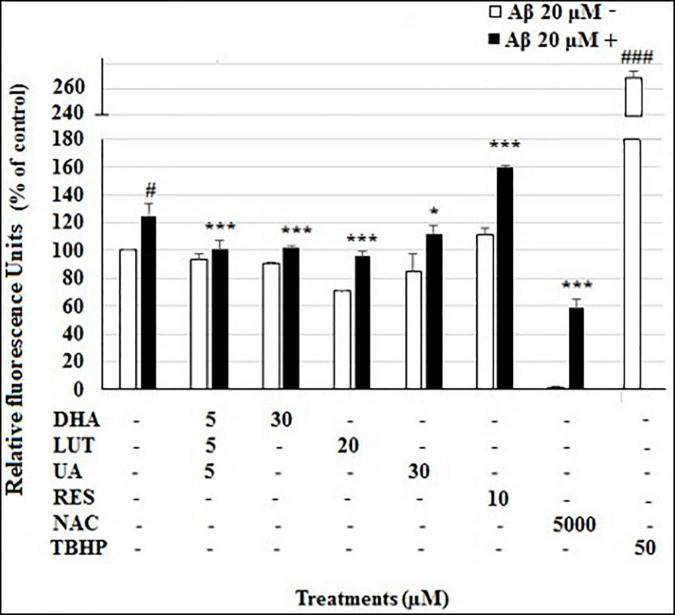
The effect of the three-compound combination and the optimal concentrations of its components against Aβ_1–42_-induced ROS levels. The BE(2)-M17 cells were pre-treated for 24 h with D_5_L_5_U_5_, LUT, DHA, and UA. Resveratrol (RES) was used at a concentration of 10 μM. Tertbutyl hydrogen peroxide (TBHP) 50 μM and NAC 5 mM were used as positive and negative controls for the assay. The cells were then exposed to 20 μM of oligomeric Aβ_1–42_ for 4 h to induce ROS production. Black bars indicate the ROS production in the presence of 20 μM Aβ_1–42_ while the white bars indicate the ROS production in the presence of 20 μM Aβ_1–42_. Cellular ROS levels were evaluated in relative fluorescence units (RFU) using the DCFDA assay. Data are expressed as mean ± SD from four (*N* = 4) independent experiments. Differences are significant at ^#^*P* < 0.05, ^###^*P* < 0.001 vs. vehicle control. *P < 0.05, ***P < 0.001vs. Aβ_1–42_-treated control.

In the DCFDA cellular ROS assay, the ROS level of vehicle control was considered as 100%. The treatment of 20 μM Aβ_1–42_ significantly increased ROS levels at 4 h to 124.8 ±4.0% (*P* < 0.05), however, D_5_L_5_U_5_, DHA, LUT, and UA significantly decreased ROS levels to 101.6 ±6.6% (*P* < 0.001), 95.7 ±4.1% (*P* < 0.001) and 111.7 ±5.9% (*P* < 0.05), respectively (Figure 1). Surprisingly however, RES resulted in further increased ROS level of 159.8 ±1.4% (*P* < 0.001). As expected, NAC significantly reduced the ROS levels to 58.4 ±6.4% (*P* < 0.001), while TBHP *per se* increased the ROS levels to 268.5 ±10.6%, compared to vehicle control (*P* < 0.001).

### D_5_L_5_U_5_, Luteolin, and Urolithin A Restores Aβ_1–42_-Induced Reduction of ATP Levels

**FIGURE 2:**
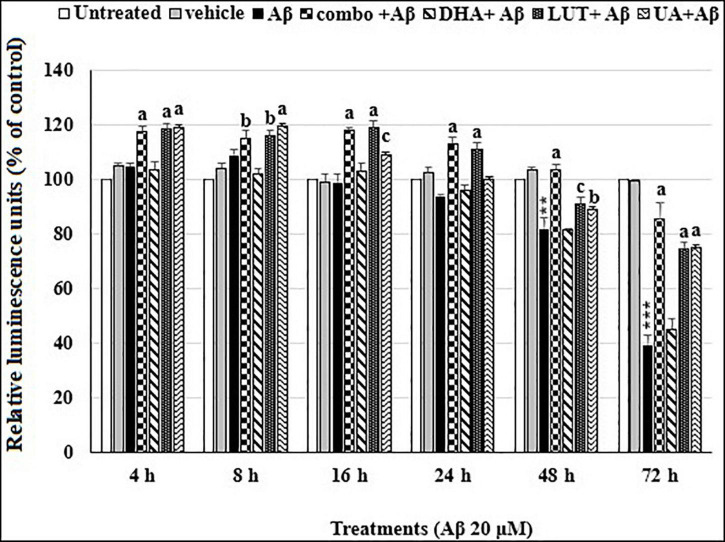
The time-dependent effects of the three-compound combination and its components on oligomeric Aβ_1–42_-affected ATP levels. The BE(2)-M17 cells were pre-treated for 24 h with D_5_L_5_U_5_, LUT, DHA, and UA. Exposure of 20 μM oligomeric Aβ_1–42_ was carried out for different time points (4–72 h) at 37°C and relative ATP levels were detected as relative luminescence units (RLU) using CellTiter Glo assay. Data are expressed as mean ± SD from four (*N* = 4) independent experiments. Differences are significant at **P = 0.001, ***P < 0.001 vs. untreated, ^b^*P* < 0.05, ^c^*P* = 0.001, ^a^*P* < 0.001 vs. Aβ_1–42_-treated controls.

As shown in Figure 2, the treatment of 20 μM Aβ_1–42_ time-dependently decreased cellular ATP levels in cells at 48 h (*P* = 0.001) and 72 h (*P* < 0.001). However, pre-treatment with D_5_L_5_U_5_, LUT, and UA caused increased ATP levels at each time point while DHA did not alter the decreased ATP levels. These results indicated that D_5_L_5_U_5_, LUT, and UA increase the ATP levels of cells exposed to Aβ_1–42_ (Figure 2).

### D_5_L_5_U_5_ Increases Mitochondrial Fusion Protein S-OPA1 and Decreases Fission Protein FIS1

**FIGURE 3:**
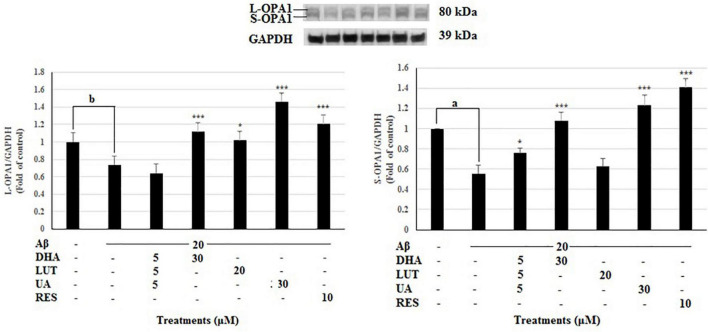
The effect of the three-compound combination and its components on the levels of L-OPA1 and S-OPA1. The BE(2)-M17 cells were pre-treated for 24 h with the three-compound combination, D_5_L_5_U_5_, DHA (30 μM), LUT (20 μM), UA (30 μM), and RES (10 μM). The cells were then exposed to 20 μM of oligomeric Aβ_1–42_ for 16 h and subjected to Western blotting. The representative immunoblot and the graph indicate the levels of L-OPA1/S-OPA1 proteins for all treatments. The relative density of the bands was normalized against GAPDH. Fold values were calculated relative to vehicle control. Data are expressed as mean ± SD from four (*N* = 4) independent experiments. Differences are significant at ^b^*P* < 0.05 vs. vehicle control, *P < 0.05, ***P < 0.001, ^a^*P* < 0.001 vs. Aβ_1–42_-treated controls.

Mitochondrial fusion proteins, OPA1 and MFN2 and the mitochondrial fission proteins FIS1 were evaluated in analyzing mitochondrial dynamics. The 20 μM Aβ_1–42_ treatment significantly decreased L-OPA1 and S-OPA1 levels (*P* < 0.05 and *P* < 0.001, respectively). However, pre-treatments with DHA, LUT, UA and RES increased L-OPA1 levels (*P* < 0.001, *P* < 0.05, *P* < 0.001, and *P* < 0.001) while D_5_L_5_U_5_, DHA, UA, and RES increased S-OPA1 levels (*P* < 0.05, *P* < 0.001, *P* < 0.001, and *P* < 0.001) (Figure 3). These results indicate that D_5_L_5_U_5_, DHA, LUT, UA, and RES all increase OPA1 levels in general.

**FIGURE 4:**
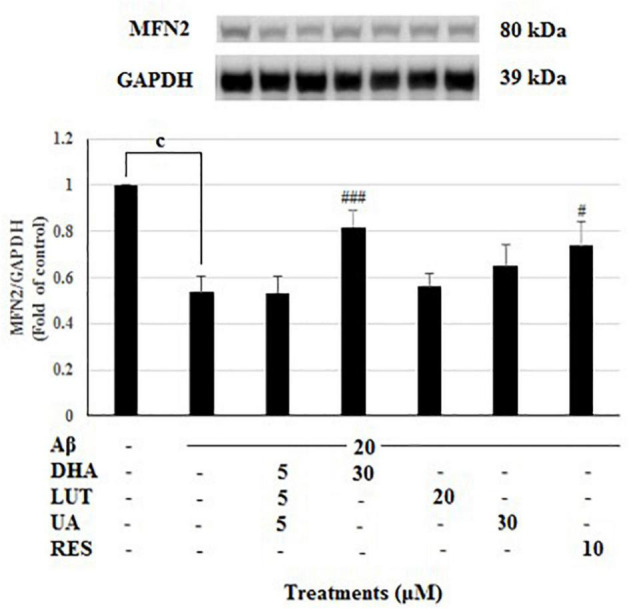
The effect of three-compound combination and its components on the levels of MFN2. The BE(2)-M17 cells were pre-treated for 24 h with the three-compound combination, D_5_L_5_U_5_, DHA (30 μM), LUT (20 μM), UA (30 μM), and RES (10 μM). The cells were then exposed to 20 μM of oligomeric Aβ_1–42_ for 16 h and subjected to Western blotting. The representative immunoblot and the graph indicate the levels of MFN2 protein for all treatments. The relative density of the bands was normalized against GAPDH. Fold values were calculated relative to vehicle control. Data are expressed as mean ± SD from four (*N* = 4) independent experiments. Differences are significant at ^c^*P* < 0.001, ^#^*P* < 0.05, ^###^*P* < 0.001 vs. Aβ_1–42_-treated control.

Treatment of 20 μM Aβ_1–42_ markedly decreased the MFN2 levels (*P* < 0.001) indicating that Aβ_1–42_ treatment inhibits the mitochondrial fusion process at 16 h, however, pre-treating with DHA and RES increased the MFN2 levels (*P* < 0.001, *P* < 0.05) while pre-treatment with D_5_L_5_U_5_ did not give any change (Figure 4).

**FIGURE 5:**
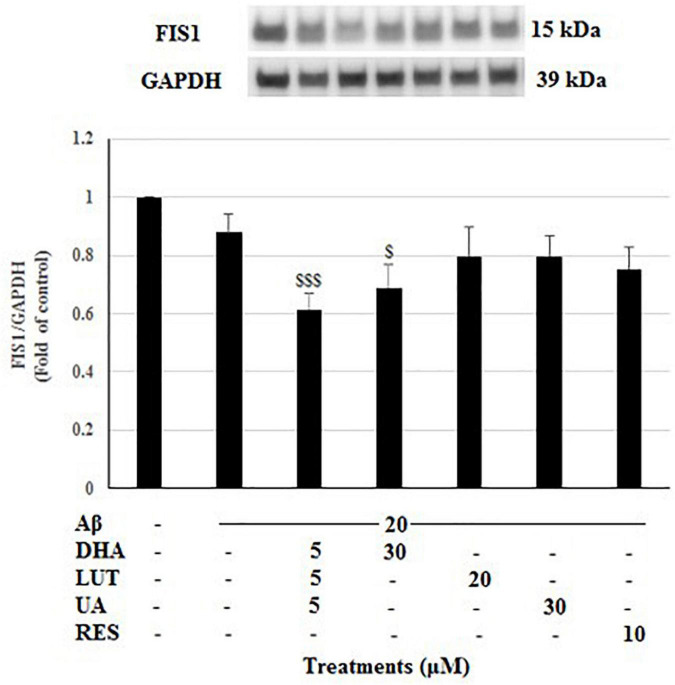
The effect of three-compound combination and its components on the levels of FIS1. The BE(2)-M17 cells were pre-treated for 24 h with the three-compound combination, D_5_L_5_U_5_, DHA (30 μM), LUT (20 μM), UA (30 μM), and RES (10 μM). The cells were then exposed to 20 μM of oligomeric Aβ_1–42_ for 16 h and subjected to Western blotting. The representative immunoblot and the graph indicate the levels of FIS1 protein for all treatments. The relative density of the bands was normalized against GAPDH. Fold values were calculated relative to vehicle control. Data are expressed as mean ± SD from four (*N* = 4) independent experiments. Differences are significant at ^$^*P* < 0.05, ^$$$^*P* < 0.001 vs. Aβ_1–42_-treated controls.

The treatment of 20 μM Aβ_1–42_ caused a reduced trend in the FIS1 levels which is indicative of reduced mitochondrial fission process. However, all the compound treatments failed to restore the FIS1 levels, instead, D_5_L_5_U_5_, and DHA resulted in significantly reduced FIS1 levels (*P* < 0.001 and *P* < 0.05) (Figure 5).

### Urolithin A, Luteolin, and DHA Induces Mitophagy and D_5_L_5_U_5_ Increases the Mitophagy Protein NDP52 but Not the Mitophagic Flux

The present study evaluated the impact of the test compounds on mitophagy to assess their activity on protecting against the Aβ_1–42_-induced effect on mitophagy. To achieve this, the expression levels of a panel of mitophagy-related proteins including LC3-I/II, p62, NDP52, OPTN, PINK1, and IMM protein TIMM23 was assessed. For p62, NDP52, OPTN, additional treatments were carried out with Bafilomycin A (Baf A)-mediated lysosomal blockade to examine the transit of the mitophagy adaptors through the autophagy pathway. Under lysosomal blockade, it has been reported by Klionsky et al. (2016), that if a lysosomal blockade of a certain treatment (with Baf A) results in increased level of a degradable autophagosomal protein (e.g., p62, NDP52, OPTN) compared to that of the treatment and the Baf A-only group, it can be considered that the selective treatment enhances autophagic flux (Klionsky et al., 2016). Similarly, if the relevant protein level of lysosomal blockade of a certain treatment is higher than that of the Baf A-only group, it can be deemed that the treatment increases synthesis of autophagy-related membranes. On the other hand, if the treatment results in lower levels of the relevant protein compared to its lysosomal blockade (with Baf A), the treatment is considered to induce a partial block in autophagic flux. If these two entities are similar, a complete blockade of autophagy at the terminal stages is expected (Klionsky et al., 2016). These factors were made use in inferring mitophagy in the present study. However, consistent results could not be obtained for LC3I/II blots.

**FIGURE 6:**
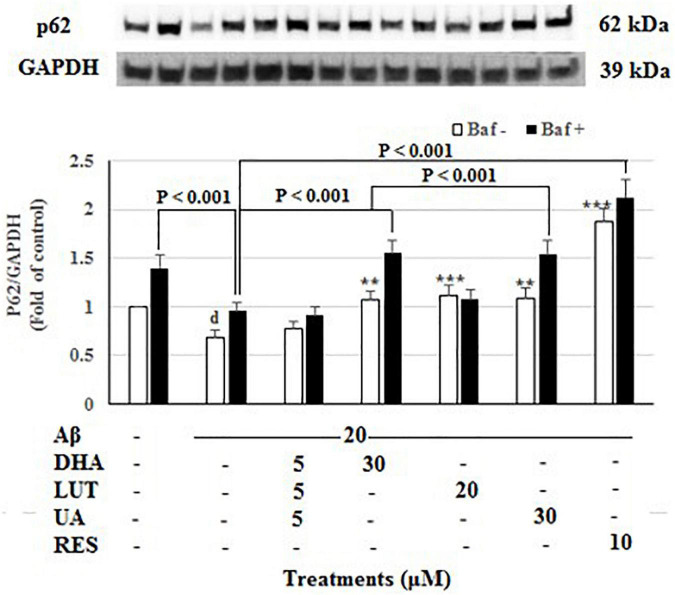
The effect of the three-compound combination and its components on the levels of p62. The BE(2)-M17 cells were pre-treated for 24 h with the three-compound combination, D_5_L_5_U_5_, DHA (30 μM), LUT (20 μM), UA (30 μM), and RES (10 μM). The cells were then exposed to 20 μM of oligomeric Aβ_1–42_ for 16 h and subjected to Western blotting. The representative immunoblot and the graph indicate the levels of p62 protein for all treatments. Black bars indicate the p62 levels in the presence of bafilomycin A (Baf A) while the white bars indicate the p62 levels in the absence of Baf A. The relative density of the bands was normalized against GAPDH. Fold values were calculated relative to vehicle control. Data are expressed as mean ± SD from four (*N* = 4) independent experiments. Differences are significant at ^d^*P* < 0.05 vs. vehicle control, **P = 0.001, ***P < 0.001 vs. Aβ_1–42_-treated control.

As shown in Figure 6, the insult of 20 μM Aβ_1–42_ for 16 h significantly decreased p62 levels (*P* < 0.05), representing a decrease in mitophagy (Klionsky et al., 2016). Furthermore, decrease of intensity in Baf A-treated Aβ_1–42_ control relative to Baf A-treated vehicle (*P* < 0.001) further confirmed that the exposure of 20 μM Aβ_1–42_ caused the increased synthesis of autophagy-related membranes. Pre-treatments with DHA, LUT, UA, and RES significantly increased p62 levels (*P* = 0.001, *P* < 0.001, *P* = 0.001 and *P* < 0.001) suggesting the induction of mitophagy. Similarly, considering the lysosomal blockade by Baf-A, p62 levels were increased by DHA, UA, and RES (*P* < 0.001 each) relative to Baf A-treated Aβ_1–42_ treatment, suggesting that pre-treatment with these compounds increased the synthesis of autophagy-related membranes (Klionsky et al., 2016).

**FIGURE 7:**
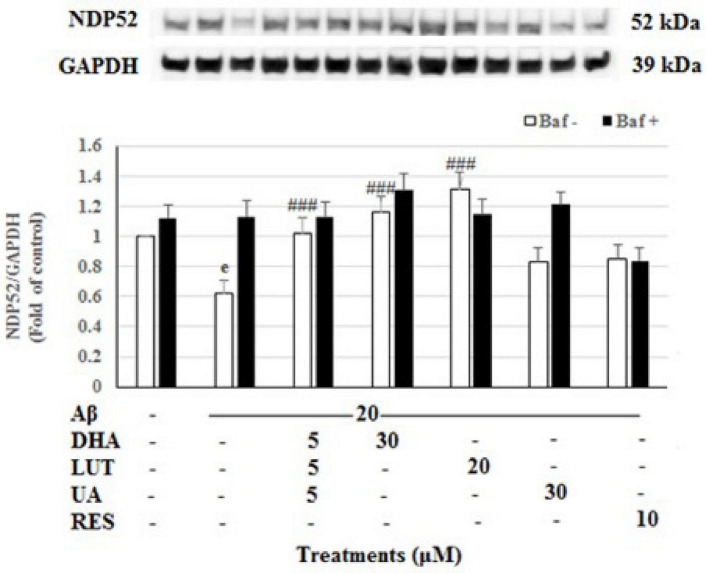
The effect of the three-compound combination and its components on the levels of NDP52. The BE(2)-M17 cells were pre-treated for 24 h with the three-compound combination, D_5_L_5_U_5_, DHA (30 μM), LUT (20 μM), UA (30 μM), and RES (10 μM). The cells were then exposed to 20 μM of oligomeric Aβ_1–42_ for 16 h and subjected to Western blotting. The representative immunoblot and the graph indicate the levels of NDP52 protein for all treatments. Black bars indicate the NDP52 levels in the presence of bafilomycin A (Baf A) while the white bars indicate the NDP52 levels in the absence of Baf A. The relative density of the bands was normalized against GAPDH. Fold values were calculated relative to vehicle control. Data are expressed as mean ± SD from four (*N* = 4) independent experiments. Differences are significant at ^e^*P* < 0.001 vs. vehicle control, ^###^*P* < 0.001 vs. Aβ_1–42_-treated controls.

The 20 μM Aβ_1–42_ treatment for 16 h significantly decreased the levels of mitophagy adaptor protein NDP52 (*P* < 0.001) indicating diminished mitophagy. However, pre-treatments with D_5_L_5_U_5_, DHA, and LUT significantly increased NDP52 levels (*P* < 0.001 each) (Figure 7) reflecting increased mitophagy by these compounds. However, for D_5_L_5_U_5_, DHA, and LUT, the NDP52 levels with the lysosomal blockade (with Baf A-treatment) were not significantly increased relative to Baf A-untreated for D_5_L_5_U_5_, DHA and LUT suggesting a possible block in autophagy/mitophagy at the terminal stages. On the other hand, the snapshot NDP52 levels for UA showing a statistically significant increase and lysosomal blockade for UA (Baf A-treated UA) resulting in higher NDP52 levels suggest that mitophagic flux is increased for UA. On the other hand, NDP52 level for RES resulting in a comparable level to that of lysosomal blockade (Baf A-treated RES) is indicative of complete block in mitophagy at the terminal stages.

**FIGURE 8:**
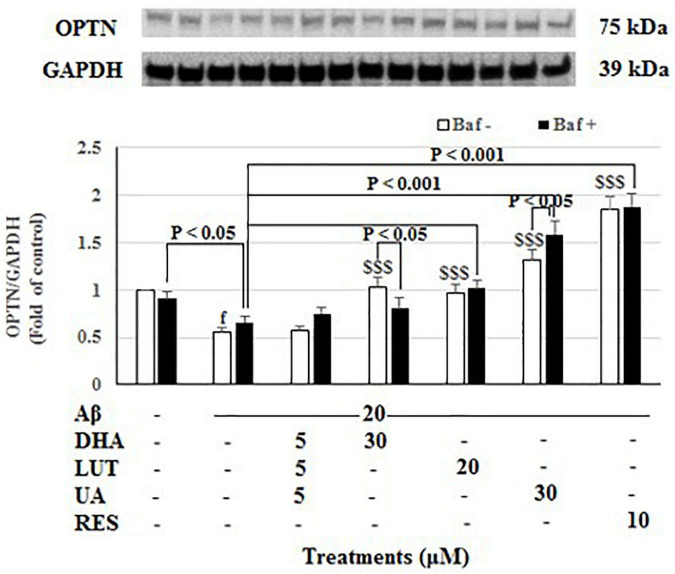
The effect of the three-compound combination and its components on the levels of OPTN. The BE(2)-M17 cells were pre-treated for 24 h with the three-compound combination, D_5_L_5_U_5_, DHA (30 μM), LUT (20 μM), UA (30 μM), and RES (10 μM). The cells were then exposed to 20 μM of oligomeric Aβ_1–42_ for 16 h and subjected to Western blotting. The representative immunoblot and the graph indicate the levels of OPTN protein for all treatments Black bars indicate the OPTN levels in the presence of bafilomycin A (Baf A) while the white bars indicate the OPTN levels in the absence of Baf A. The relative density of the bands was normalized against GAPDH. Fold values were calculated relative to vehicle control. Data are expressed as mean ± SD from four (*N* = 4) independent experiments. Differences are significant at ^f^*P* < 0.001 vs. vehicle control, ^$$$^*P* < 0.001 vs. Aβ_1–42_-treated control.

The treatment of 20 μM Aβ_1–42_ for 16 h significantly decreased OPTN levels (*P* < 0.001) indicating decreased mitophagy. Furthermore, decreasing of Aβ_1–42_ control with lysosomal blockade (Baf A-treated) relative to Baf A-treated vehicle (*P* < 0.05) further confirmed that exposure of 20 μM Aβ_1–42_ decreased the synthesis of autophagy-related membranes. Pre-treatments with DHA, LUT, UA, and RES significantly increased OPTN levels (*P* < 0.001 each) reflecting an induction of mitophagy. Moreover, considering lysosomal blockade, increased OPTN levels for Baf A-treated UA (*P* < 0.05) suggested increased synthesis of autophagy-related membranes. However, RES and LUT resulting in unaltered OPTN levels for Baf A-treatments suggest a complete block of autophagy/mitophagy at the terminal stages. DHA resulting in decreased OPTN for Baf A-treatment (*P* < 0.05 each) implicated that a partial block in autophagic flux is induced by DHA (Figure 8).

**FIGURE 9:**
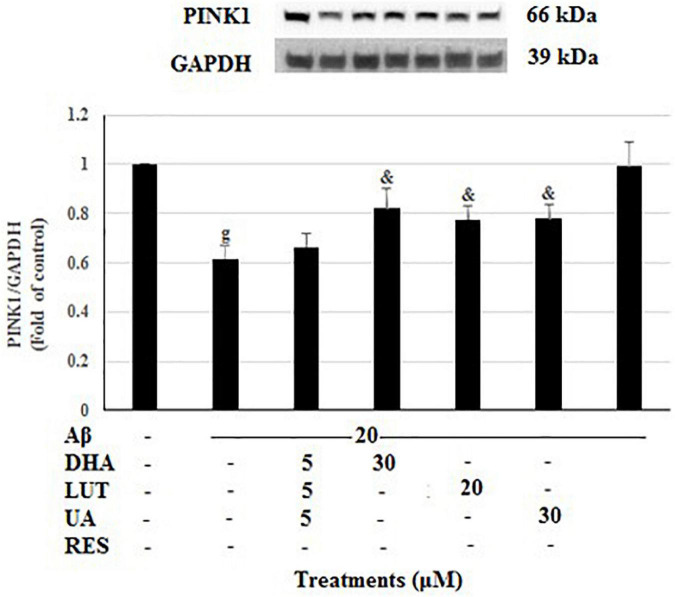
The effect of the three-compound combination and its constituents on the levels of PINK1. The BE(2)-M17 cells were pre-treated for 24 h with the three-compound combination, D_5_L_5_U_5_, DHA (30 μM), LUT (20 μM), UA (30 μM), and RES (10 μM). The cells were then exposed to 20 μM of oligomeric Aβ_1–42_ for 16 h and subjected to Western blotting. The representative immunoblot and the graph indicate the levels of PINK1 protein for all treatments. The relative density of the bands was normalized against GAPDH. Fold values were calculated relative to vehicle control. Data are expressed as mean ± SD from four (*N* = 4) independent experiments. Differences are significant at ^g^*P* < 0.001 vs. vehicle control, ^&^*P* < 0.05 vs. Aβ_1–42_-treated control.

As shown in Figure 9, 20 μM Aβ_1–42_ treatment markedly decreased PINK1 levels (*P* < 0.001) suggesting that Aβ_1–42_ causes a mitophagy deficit in PINK1/Parkin-mediated mitophagy pathway. However, pre-treatments with DHA, LUT, UA, and RES increased the PINK1 levels (*P* < 0.05, *P* < 0.05, *P* < 0.05, and *P* < 0.001). These results support the notion that DHA, LUT, UA, and RES may induce mitophagy possibly through PINK1/Parkin mediated pathway.

**FIGURE 10:**
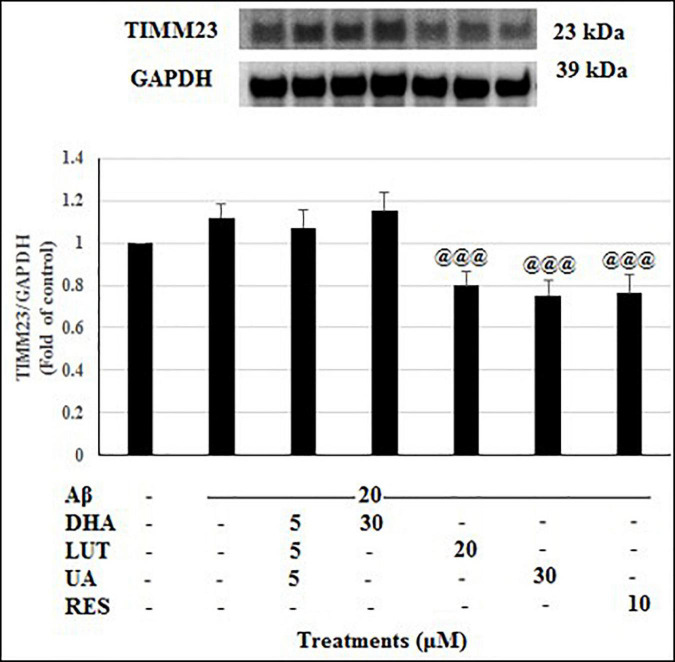
The effect of the three-compound combination and its components on the levels of TIMM23. The BE(2)-M17 cells were pre-treated for 24 h with the three-compound combination, D_5_L_5_U_5_, DHA (30 μM), LUT (20 μM), UA (30 μM), and RES (10 μM). The cells were then exposed to 20 μM of oligomeric Aβ_1–42_ for 16 h and subjected to Western blotting. The representative immunoblot and the graph indicate the levels of TIMM23 protein for all treatments. Semi-quantitative analysis of TIMM23 protein levels. The relative density of the bands was normalized against GAPDH. Fold values were calculated relative to vehicle control. Data are expressed as mean ± SD from four (*N* = 4) independent experiments. Differences are significant at ^@@@^*P* < 0.001 vs. Aβ_1–42_-treated control.

The treatment of 20 μM Aβ_1–42_ for 16 h showed no significant difference in the TIMM23 levels compared to the untreated. Considering the TIMM23 levels as a marker of number of mitochondria, the stable TIMM23 levels suggest three possibilities. First, reduced mitophagy and mitobiogenesis. Second, an increase of both processes and thirdly, no change is both the processes. Similarly, the pre-treatments with D_5_L_5_U_5_ and DHA did not alter the levels of TIMM23 (Figure 10). On the other hand, LUT, UA and RES markedly decreased the TIMM23 levels (*P* < 0.001 each) when compared to the Aβ_1–42_-treated control. A reduction in TIMM23 levels can be a consequence of enhanced mitophagy or impairments in the mitochondrial biogenesis. Therefore, for further inference, the effects of the compounds on mitobiogenesis were evaluated next.

### D_5_L_5_U_5_ Increases Mitochondrial Biogenesis-Related Protein PGC1-α

**FIGURE 11:**
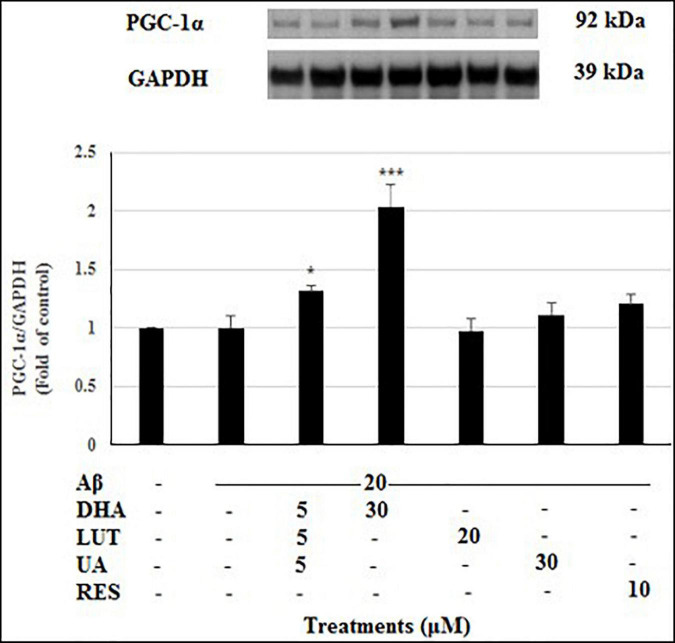
The effect of three-compound combination and its components on the levels of PGC1-α. The BE(2)-M17 cells were pre-treated for 24 h with the three-compound combination, D_5_L_5_U_5_, DHA (30 μM), LUT (20 μM), UA (30 μM), and RES (10 μM). The cells were then exposed to 20 μM of oligomeric Aβ_1–42_ for 16 h and subjected to Western blotting. The representative immunoblot and the graph indicate the levels of PGC1-α protein for all treatments. The relative density of the bands was normalized against GAPDH. Fold values were calculated relative to vehicle control. Data are expressed as mean ± SD from four (*N* = 4) independent experiments. Differences are significant at *P < 0.05, ***P < 0.001 vs. Aβ_1–42_-treated control.

Mitobiogenesis was evaluated in the present study by quantifying PGC1-α. As shown in Figure 11, exposure of 20 μM Aβ_1–42_ for 16 h did not alter the levels of PGC1-α indicating that mitobiogenesis is not affected. However, pre-treatment with D_5_L_5_U_5_ (*P* < 0.05) and DHA elevated PGC1-α levels with the latter resulting in a marked increase (*P* < 0.001) reflecting their increased ability to induce mitobiogenesis. These results taken together with the findings of altered TIMM23 levels (Figure 10) provide some insight of these compounds on mitophagy.

The results summarized in the Table 1 shows that after 16 h of Aβ_1–42_ exposure, the dynamics-related proteins L-OPA1, S-OPA1, MFN2, and FIS1 and the mitophagy proteins, p62, OPTN, and PINK1 all decreased. These results, along with the increased TIMM23 levels at 16 h indicate that there is a deficit in mitophagy in response to 16 h Aβ_1–42_ exposure. However, the three-compound combination resulted in increased NDP52/PINK1 and PGC-1α levels indicating its ability to induce mitophagy and mitobiogenesis, respectively. Treatment with DHA increased OPA1 levels, p62/NDP52/OPTN/PINK1 levels and PGC-1α, indicating its activity in increasing mitochondrial fusion, mitophagy and mitobiogenesis. LUT resulted in increased L-OPA1 and p62/NDP52 levels reflecting its ability to induce mitochondrial fusion and mitophagy, respectively. Finally, UA was shown to induce mitochondrial fusion and mitophagy due to increased OPA1 levels and p62/OPTN levels, respectively.

**TABLE 1:** Changes in mitochondrial dynamics-related proteins, mitophagy-related proteins, and the mitobiogenesis-related protein with pre-treatment of compounds followed by Aβ_1–42_ treatment.

Proteins	Aβ_1–42_-control	D_5_L_5_U_5_	DHA (30 μM)	LUT (20 μM)	UA (30 μM)	RES (10 μM)
**For all proteins, vehicle-treated control was considered as 1**						
**Mitochondrial dynamics-related proteins**						
L-OPA1	-	–	+	+	+++	+
S-OPA1	–	-	+	–	++	++
MFN2	–	–	-	–	–	-
FIS1	-	–	–	-	-	-
**Mitophagy-related proteins**						
p62	–	-	+	++	+	+++
NDP52	–	+	+	++	-	-
OPTN	–	–	+	-	++	+++
PINK1	–	–	-	-	-	-
TIMM23	+	+	+	-	-	-
**Mitochondrial biogenesis-related protein**						
PGC-1α	-	++	+++	-	+	++

*Using Western blotting, protein fold changes were assessed in BE(2)-M17 cells pre-treated with D_5_L_5_U_5_, DHA, LUT, UA, and RES for 24 h followed by 20 μM oligomeric Aβ_1–42_ exposure for 16 h. ± values indicate the relative protein fold change compared to vehicle controls.*

*Protein fold changes at 0.50–0.70: –, 0.71–0.99: -, 1.01–1.20: +, 1.21–1.40: ++, 1.41 upwards: +++.*

*Significant changes vs. Aβ_1–42_-treated control are indicated by 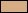
*

## Discussion

Evidence is accumulating that a healthy diet enriched with bioactive components have a protective role in reducing the risk of AD. The current study presented a comprehensive cell-based investigation of the therapeutic effects of a previously determined nutraceutical combination, D_5_L_5_U_5_ (Jayatunga et al., 2021) against Aβ_1–42_-induced toxicity. As per the initial test results of MTS and ATP assays, the combination was anticipated to have beneficial effects on mitochondria. On the other hand, mitochondrial dysfunction is an important feature in Aβ_1–42_-induced toxicity (Chen and Yan, 2007). Dysfunctional mitochondria are often portrayed by increased oxidative stress and deficits in mitochondrial dynamics, mitophagy and mitochondrial biogenesis. Therefore, inducing these processes are important therapeutic targets in AD (Su et al., 2010; Uittenbogaard and Chiaramello, 2014; Jayatunga et al., 2020). In this light, the present study explored the neuroprotective action of D_5_L_5_U_5_.

In AD, excessive ROS is generated from impaired mitochondria as well as through Aβ interactions with transition metal ions. In fact, abnormal accumulation of Aβ and Tau proteins promote redox imbalance leading to oxidative stress. However, existing antioxidants have been shown to be relatively ineffective in combating AD (Lloret et al., 2011; Lloret et al., 2019). There may be several reasons for this. First, the antioxidants may not be able to cross the BBB and reach the specific sites of ROS release in the brain, which are suggested to be the hydrophobic domains within mitochondrial membranes. Second, the antioxidants may have a poor ability to scavenge ROS. The present study used the DCFDA assay to determine ROS in the cells. The DCFDA reagent diffuses into cells and gets deacetylated by cellular esterases to a non-fluorescent compound that gets oxidized to a fluorescent compound by cellular ROS. The results revealed that D_5_L_5_U_5_ as well its components, DHA, LUT, and UA, decreased the cellular ROS levels, generated by 4 h of exposure of 20 μM Aβ_1–42_. The potency of the antioxidant activity was, LUT > D_5_L_5_U_5_ > DHA > UA (Figure 1).

Luteolin showed the highest antioxidant activity in the present study (Figure 1). It is consistent with previous reports indicating antioxidant effects of LUT (Xiao et al., 2019). Furthermore, it has been shown that LUT induces apoptosis by promoting antioxidant activity and activating MAPK signaling in human colon cancer cells (Kang et al., 2017). Being a flavone, the antioxidant activity of LUT is thought to possibly be exerted by its chemical structure consisting of four phenolic hydroxyl groups and C2=C3 double bond in conjugation with the C4-carbonyl group (Cai et al., 2006; Vo et al., 2019). Docosahexaenoic acid being a PUFA, showed a moderate ability reduce cellular ROS levels in the present study, and hence, DHA may act as a lipophilic antioxidant that can reach the mitochondrial hydrophobic domains of ROS production in the brain. Despite being a highly oxidizable polyunsaturated fatty acid, DHA is concentrated as a naturally occurring omega-3-PUFA in neuronal brain membranes where approximately 30–50% of total dry weight of the brain is lipid. In fact, there are reports of DHA on enzymatic antioxidant activities, such as catalase, glutathione peroxidase (GPx) and glutathione reductase (GR) (Hossain et al., 1999; Hashimoto et al., 2005).

The second highest potency of antioxidant activity was observed with the D_5_L_5_U_5_ combination (Figure 1) accounting for its ability to inhibit Aβ_1–42_-induced toxicity effectively (Jayatunga et al., 2021). The data showed that although UA could reduce ROS, it offered the least protection compared to other compounds. Previous work of UA has shown that it can decrease intracellular ROS levels and increase intracellular SOD and GPx activity *in vitro* (Wang et al., 2015). However, UA has been shown to exert pro-oxidant activities as well (Kallio et al., 2013). Moreover, at higher concentrations, a bi-phasic effect of DHA has also been reported in human blood platelets due to its pro-oxidant effects (Vericel et al., 2003). Luteolin is also reported to act as a pro-oxidant as part of its redox modulation activity (Lin et al., 2008). Thus, the three-compound combination, D_5_L_5_U_5_ may also potentially have a pro-oxidant activity, however, it was not evaluated in the present study. It must be noted that the redox properties of compounds are highly reliant on the reaction conditions and employed assay system. Hence, it is important to study both the pro-oxidant and antioxidant activities simultaneously if possible (Kallio et al., 2013).

Apart from the above-mentioned ROS scavenging antioxidant strategies, the ability to directly block mitochondrial ROS production may be an effective way of inhibiting oxidative stress. Whist, it seems challenging to define both the normal and pathologically relevant ROS formation, lowering of ROS production by efficiently stabilizing mitochondrial energy production appears feasible. In healthy mitochondria, ATP, and ROS levels are coupled. However, it has been reported that a mild mitochondrial uncoupling (resulting in increased ATP levels and decreased ROS levels) due to controlled reduction of MMP, is a highly effective antioxidant strategy (Caldeira da Silva et al., 2008).

Therefore, the current study was designed to assess the ability of the compounds to restore the ATP levels affected by the administration of Aβ_1–42_. As assessed at 4–72 h of Aβ_1–42_ exposure, the reduced ATP levels are increased by pre-treatment with the compounds. Considering the activity of all the time points, the overall order of potency of increasing the ATP production by the compounds was, D_5_L_5_U_5_ > LUT > UA (Figure 2). This order of potency was consistent over every time point. The mechanism by which ATP levels were increased is presently unclear, however the speculation is that the compounds could accept electrons from oxidative species and transfer them to cytochrome c, bypassing respiratory complexes I and III, at a level sufficient to compensate for reduced proton pumping (Komlódi and Tretter, 2017; Berry et al., 2018; Zhao et al., 2019).

Mitochondrial spare respiratory capacity is known to be an important aspect of mitochondrial function and is defined as the difference between basal ATP production and its maximal activity (Deng et al., 2015; Pfleger et al., 2015). It accounts for the extra ATP that can be produced by OXPHOS to overcome various cellular stressors. A cell with a larger spare respiratory capacity can produce more ATP and, in theory, overcome more stress. It has been reported that various polyphenolic compounds can augment the mitochondrial spare respiratory capacity by increasing mitochondrial biogenesis and antioxidant activities (Yamamoto et al., 2016). Although it was compelling to verify this effect for the compounds, in the present study itself, the requirement of bioenergetic flux analyses to confirm whether the compounds promoted enhanced oxygen consumption made it beyond the scope of the study.

It has been shown in our previous studies that the time of exposure of Aβ_1–42_ to cells is a crucial determinant for inducing significant detrimental effects on mitochondrial dynamics, mitophagy and mitobiogenesis (Jayatunga et al., unpublished). Therefore, 20 μM Aβ_1–42_ exposure time of 16 h was selected to determine the time-dependent marked deficits of mitochondrial dynamics, mitophagy and mitobiogenesis. Mitochondrial dynamics-related proteins (OPA1, MFN2, and FIS1), mitophagy-related proteins (p62, NDP52, OPTN, PINK1), IMM protein TIMM23 and mitochondrial biogenesis protein PGC-1α were analyzed using western blotting. Resveratrol was used as a reference compound for its ability to induce mitophagy (Kuno et al., 2018; Cao et al., 2019) and mitobiogenesis (Csiszar et al., 2009).

In the present study, DHA was shown to have increased L-OPA1, S-OPA1, and MFN2 levels implying its effects on increasing mitochondrial fusion. The increased TIMM23 levels suggest that DHA may inhibit mitophagy or promote biogenesis. However, DHA induced mitophagy as well as mitobiogenesis due to its ability to increase of p62, OPTN, PINK1 and PGC-1α. Therefore, the increased TIMM23 levels denote the excess mitochondria biosynthesized after compensation with mitophagy. These findings are consistent with the previous studies that have identified the role of DHA as a potential mitophagy or autophagy inducer, or both (Jing et al., 2011; Shin et al., 2013).

It has been reported that mitobiogenesis is related to sterol regulatory element (SRE)-binding protein (SREBP-1c) as it is implicated in upregulation of PGC-1α (Kobayashi et al., 2018). However, it has been reported that DHA inhibits the promoter activity of SREBP-1c (Deng et al., 2015; Huang et al., 2017) suggesting that DHA may not promote mitobiogenesis in that pathway. However, the present study resulted in an outcome contrary to the above-mentioned studies by showing DHA induced mitobiogenesis. However, the current findings are consistent with several other reports that indicate mitobiogenesis is induced by DHA (Flachs et al., 2005; Lee et al., 2016).

Luteolin also appears to act as an autophagy/mitophagy inducer based on the present findings, due to increased p62, OPTN, PINK1 levels and decreased TIMM23 levels. It did not exert beneficial effects in inducing mitobiogenesis, despite inducing mitochondrial fusion due to increased L-OPA1 levels. These findings are in line with previous reports indicating that LUT is an autophagy or mitophagy inducing compound (Hu et al., 2016). In contrary to the present study, it has also been reported that LUT inhibited autophagy (Li et al., 2019) and induced mitobiogenesis through inhibition of the Mst-1 pathway (Hu et al., 2016).

The present study revealed that UA induced mitophagy as indicated by increased expression levels of p62, NDP52, OPTN, and PINK1. The OPA1 levels were also increased by UA treatment, indicating increased mitochondrial fusion. However, UA only exhibited a non-significant trend for raised PGC-1α levels, indicating that mitobiogenesis was not occurring at detectable levels. These findings of UA on inducing mitophagy are in line with previous reports (Ryu et al., 2016; Webb, 2017). Moreover, UA has shown to be beneficial in both Aβ and Tau based *Caenorhabditis elegans* models of AD (Fang et al., 2019). While one report states that UA stimulates autophagy but not mitophagy to inhibit ER stress in a model of ischemic neuronal injury (Ahsan et al., 2019), our findings indicated that UA induced both autophagy and mitophagy.

The present study revealed that pre-treatment with D_5_L_5_U_5_ increased NDP52 (*P* < 0.001), an autophagy adaptor protein related to mitophagy, revealing its ability to trigger mitophagy. In fact, D_5_L_5_U_5_ increased PGC-1α levels (*P* < 0.05) indicating induction of mitobiogenesis. The increased trend of TIMM23 was attributed to the mitochondrial mass balanced by mitobiogenesis and mitophagy processes. It was also indicated that D_5_L_5_U_5_ increased S-OPA1 levels (*P* < 0.05). Apart from its action in mitochondrial fission, as a prerequisite to mitophagy, S-OPA1 has also been proposed to act as an enhancer of OPA1-Cardiolipin interaction that facilitates mitochondrial fusion (Rujiviphat et al., 2009). This may explain how the three-compound combination, D_5_L_5_U_5_, increased mitochondrial fusion to result in increased mitobiogenesis.

The order of potency for mitophagy-inducing activity of the compounds is UA > DHA > LUT > D_5_L_5_U_5_. However, the utility of inducing mitophagy is undoubtedly preferred only in a controlled manner without causing deterioration or toxicity in cellular respiration. In this regard, these compounds also have strong antioxidant properties. Hence, it is obvious that their mechanisms of activating mitophagy are not ROS-dependent or mitohormetic, thus positioning them as important candidates for potential AD therapy. Moreover, considering the antioxidant properties determined through the cellular ROS levels, the compounds can be positioned as UA > DHA > D_5_L_5_U_5_ > LUT, suggesting a correlation between the level of ROS and mitophagy only at higher levels of ROS. It has been speculated that mitochondrial ROS (superoxide) involves in mitophagy, but this notion remains to be verified (Shefa et al., 2019; Schofield and Schafer, 2020). Further studies are needed to investigate the molecular mechanisms by which the three-compound combination reduces ROS levels and induces mitophagy and mitobiogenesis.

Compounds that activate mitophagy without inducing a respiration collapse are important as protectors of mitochondria. The three-compound combination, D_5_L_5_U_5_ effectively attenuated Aβ_1–42_-induced toxicity (Jayatunga et al., 2021), possibly due its ability to decrease ROS levels, induce mitobiogenesis and mitophagy, to some extent. In fact, D_5_L_5_U_5_ was shown to have its protective ability on all aspects tested, namely, by reducing ROS levels, increasing ATP levels, and inducing mitophagy and mitobiogenesis. Taken together, having DHA, LUT, and UA in one single entity as D_5_L_5_U_5_ immensely benefits and precedes the mitochondrial protective role as shown in the current study. It is of note that there may be other pathways that may account for the neuroprotection exerted by this synergistic nutraceutical combination. Further *in vitro* and *in vivo* studies are required to uncover the specific modes of mitophagy and mitobiogenesis elicited by the D_5_L_5_U_5_.

## Data Availability Statement

The raw data supporting the conclusions of this article will be made available by the authors, without undue reservation.

## Author Contributions

RM and DJ designed the study. DJ wrote the manuscript. RM, EH, GV, MG, and WF reviewed the manuscript intensively. MG and WF edited the manuscript. All authors have read and agreed to the final version of this manuscript.

## Conflict of Interest

The authors declare that the research was conducted in the absence of any commercial or financial relationships that could be construed as a potential conflict of interest.

## Publisher’s Note

All claims expressed in this article are solely those of the authors and do not necessarily represent those of their affiliated organizations, or those of the publisher, the editors and the reviewers. Any product that may be evaluated in this article, or claim that may be made by its manufacturer, is not guaranteed or endorsed by the publisher.
